# Ultra-effective modified clinoptilolite adsorbent for selective thorium removal from radioactive residue

**DOI:** 10.1038/s41598-023-36487-5

**Published:** 2023-06-08

**Authors:** Abdulrahman Masoud Alotaibi, Aznan Fazli Ismail, Eli Syafiqah Aziman

**Affiliations:** 1 Nuclear Science Programme, Faculty of Science and Technology, Universiti Kebangsaan Malaysia, UKM, 43600 Bangi, Selangor Malaysia; 2 Department of Physics, Faculty of Applied Science, Umm Al-Qura University, Makkah, 21955 Saudi Arabia; 3 Nuclear Technology Research Centre, Faculty of Science and Technology, Universiti Kebangsaan Malaysia, UKM, 43600 Bangi, Selangor Malaysia

**Keywords:** Environmental sciences, Materials science

## Abstract

This study investigated the efficacy of using phosphate-modified zeolite (PZ) as an adsorbent for removing thorium from aqueous solutions. The effects of various factors such as contact time, adsorbent mass, initial thorium concentration, and pH value of the solution on the removal efficiency were analyzed using the batch technique to obtain optimum adsorption condition. The results revealed that the optimal conditions for thorium adsorption were a contact time of 24 h, 0.03 g of PZ adsorbent, pH 3, and a temperature of 25 °C. Isotherm and kinetics parameters of the thorium adsorption on PZ were also determined, with equilibrium studies showing that the experimental data followed the Langmuir isotherm model. The maximum adsorption capacity (Q_o_) for thorium was found to be 17.3 mg/g with the Langmuir isotherm coefficient of 0.09 L/mg. Using phosphate anions to modify natural zeolite increased its adsorption capacity. Furthermore, adsorption kinetics studies demonstrated that the adsorption of thorium onto PZ adsorbent fitted well with the pseudo-second-order model. The applicability of the PZ adsorbent in removing thorium from real radioactive waste was also investigated, and nearly complete thorium removal was achieved (> 99%) from the leached solution obtained from cracking and leaching processes of rare earth industrial residue under optimized conditions. This study elucidates the potential of PZ adsorbent for efficient removal of thorium from rare earth residue via adsorption, leading to a reduction in waste volume for ultimate disposition.

## Introduction

Thorium is a naturally occurring radioactive element that is abundant in the Earth's crust. Although classified as a fertile element, thorium can be converted to fissile material uranium-233 through neutron bombardment and beta decay^1^. In recent years, thorium and its oxides have gained increased attention due to their numerous industrial applications, including high-quality optics, catalysis, high-temperature ceramics, nuclear medicine, and nuclear fuel industry^2–5^. The use of thorium in the nuclear fuel industry is advantageous due to its low operating cost, safe and clean energy production, and minimal generation of radioactive waste compared to uranium reactors^1,2,6,7^.

Thorium can be released into the environment from natural and human activities. Natural sources include cosmic rays, earth’s crust, and volcano eruptions, while anthropogenic sources include coal combustion, nuclear fuel plants, and ore mining and refining^8–10^. Among human activities, rare earth extraction generates a significant amount of waste containing thorium, with Malaysia being one of the countries facing this challenge due to its rare-earth processing plant that produces over 450,000 tonnes of waste with high thorium concentrations. Since the thorium content of these waste exceeds the limit of 1000 Bq/kg, the Malaysian government has classified these waste as radioactive waste^11–13^. The presence of thorium in radioactive waste is a pivotal issue in radioactive waste management because of its high radiotoxicity, chemical toxicity, and long half-life, which poses a significant threat to the environment and human health^14^. Therefore, appropriate treatment to remove thorium from radioactive waste is necessary to reduce the amount of waste that requires disposal, leading to lower disposal costs and improved disposal site efficiency.

Numerous techniques have been developed to remove thorium from radioactive wastes, including ion exchange, chemical precipitation, adsorption, and evaporation^15,16^. Among these techniques, adsorption is preferred over others, as it is safer, easier, more environmentally friendly, and cost-effective. Therefore, it has been extensively utilized for the removal of radioactive ions from waste^17–19^. Various types of adsorbents have been used for thorium removal, including zeolite^20^, bentonite^21^, glauconite^22^, chitosan^23^, and illite^24^. Among these adsorbents, natural zeolites have received significant attention from researchers due to their excellent ion exchange, catalytic, molecular sieve, and adsorption properties since the discovery of zeolites in 1756^25,26^.

The natural zeolite has been a topic of interest since the late 1950s, specifically in the treatment of low-level radioactive waste. However, it has recently been applied to the treatment of medium and high-level radioactive wastes as well^27^. Natural zeolite has several unique features, such as being inexpensive, abundant, selective, and highly stable against alpha, beta, and gamma irradiations. As a result, zeolites have become the most widely used adsorbents for treating radioactive wastewater. Natural zeolite is a crystalline aluminosilicate with a three-dimensional framework of tetrahedral alumina (AlO_4_) and silica (SiO_4_), connected by shared oxygen atoms. There are around 40 types of natural zeolites known around the world. Natural zeolites such as clinoptilolite, mordenite, phillipsite, chabazite, analcime, stilbite, and laumontite are very common types^28^. Among the recognized types of natural zeolites, clinoptilolite is one of the most significant and abundant type of natural zeolites. It is found in big deposits all over the world and is commonly used in various industrial wastewater sorption treatment research on a global scale^29,30^. Clinoptilolite is a silica-rich member of the heulandite group of natural zeolites that occurs in zeolitic volcanic tuffs^14^. The availability, favorable ion exchange capacity, and low cost of clinoptilolite make it ideally suited to be utilized as adsorbent material for the removal of hazardous metal ions^31,32^.

Natural adsorbent materials have the benefit of availability, but they suffer from low adsorption capacity, sensitivity, selectivity, and durability^33^. To address this issue, many researchers have explored methods to modify natural zeolite surfaces to enhance its adsorption effectiveness. In terms of cation exchange capacity and adsorption performance, modified zeolites are more effective and efficient than natural zeolites^34–36^. While several investigations on zeolite modification have been carried out to enhance its adsorption effectiveness for the removal of thorium^37,38^, this study represents the first scientific attempt to employ phosphate-modified zeolite for the removal of thorium from rare earth industrial residue.

The present research aims to determine the optimum experimental condition at various parameter including contact time, the mass of the adsorbent, the initial concentration of thorium, and the pH of the solution on the adsorption of thorium ions by phosphate-modified zeolite (PZ), which we did not determine in our previous work^39^. To investigate the applicability of the PZ adsorbent in removing thorium from real radioactive waste, as well as to give an idea of converting the massive quantity of radioactive waste into manageable quantities.

## Materials and methods

### Materials

In this study, natural zeolite (clinoptilolite) was used as the adsorbent material, which was purchased from Heiltropfen (Heiltropfen Lab. LPP, 27 Old Gloucester Street, WC1N 3AX, London, United Kingdom). The product is a 100% natural volcanic mineral from Slovakia, EU, with over 90% clinoptilolite content. For the surface modification of natural zeolite, potassium dihydrogen phosphate (KH_2_PO_4_, ≥ 99.0%, Mw = 136.09 g/mol) obtained from Sigma Aldrich was used. The stock standard solution of 1000 mg/L of thorium nitrate [Th(NO_3_)_4_] was purchased from AccuStandard (ICP-61N-5, New Haven-CT, USA), and various concentrations of thorium solution were prepared from the stock solution through appropriate dilution. Sulphuric acid (H_2_SO_4_, 98%) from J.T.Baker (Avantor Performance Materials, Center Valley, PA, USA) was used in the digestion process, and dilute solutions of sodium hydroxide (NaOH) and nitric acid (HNO_3_) were used to adjust the pH to the required value. All materials and chemicals were used as obtained from the suppliers without any additional purification and modification, unless otherwise stated. Throughout the experiments, deionized water was used.

### Instrumentations

The identification of the structure, purity, and crystallinity of phosphate-modified zeolite have been determined by using X-ray diffraction (XRD) (Brand: Bruker AXS Germany, Model: D8 Advance, fitted with a scintillation counter). The specific surface area (S_BET_) and other textural properties of phosphate-modified zeolite have been determined by using a Micromeritics Accelerated Surface Area and Porosimetry analyzer system (ASAP 2020, Micromeritics, Atlanta, USA). Fourier transform infrared spectroscopy (Perkin Elmer, spectrum 400 FT-IR NIR spectrometer) has been used to identify the functional groups that are present in the phosphate-modified zeolite. The surface morphology of the adsorbent material has been observed by using the field emission scanning electron microscopy (FESEM, Merlin ZEISS GEMINI 2, Oberkochen, Germany). The field emission scanning electron microscopy is equipped with energy dispersive X-ray spectroscopy (EDX) to identify the composition of the elements present in the phosphate-modified zeolite. Inductive Coupled Plasma-Mass Spectrometry (ICP-MS, Perkin Elmer Sciex Elan 9000, USA) has been used in this research to determine the equilibrium concentration of thorium remaining in the solutions after the adsorption processes. A pH meter (model EUTECH ph700; Thermo Scientific) has been used for pH measurements. A shaker (high-speed microplate shaker, Illumina, Model: 945,190, San Diego, California, USA) was used to shake the samples at 775 rpm. Centrifugation (Model: TGL-16 C centrifuge; Shanghai Longyue Instrument Equipment Co., Ltd., Shanghai, China) was employed to centrifuge the samples. In the modification process, phosphate-modified zeolite has been filtered off by using Whatman™ qualitative filter papers (Whatman’s No. 1: GE Healthcare Life Science, United Kingdom).

### Preparation of phosphate-modified zeolite

**Figure 1:**
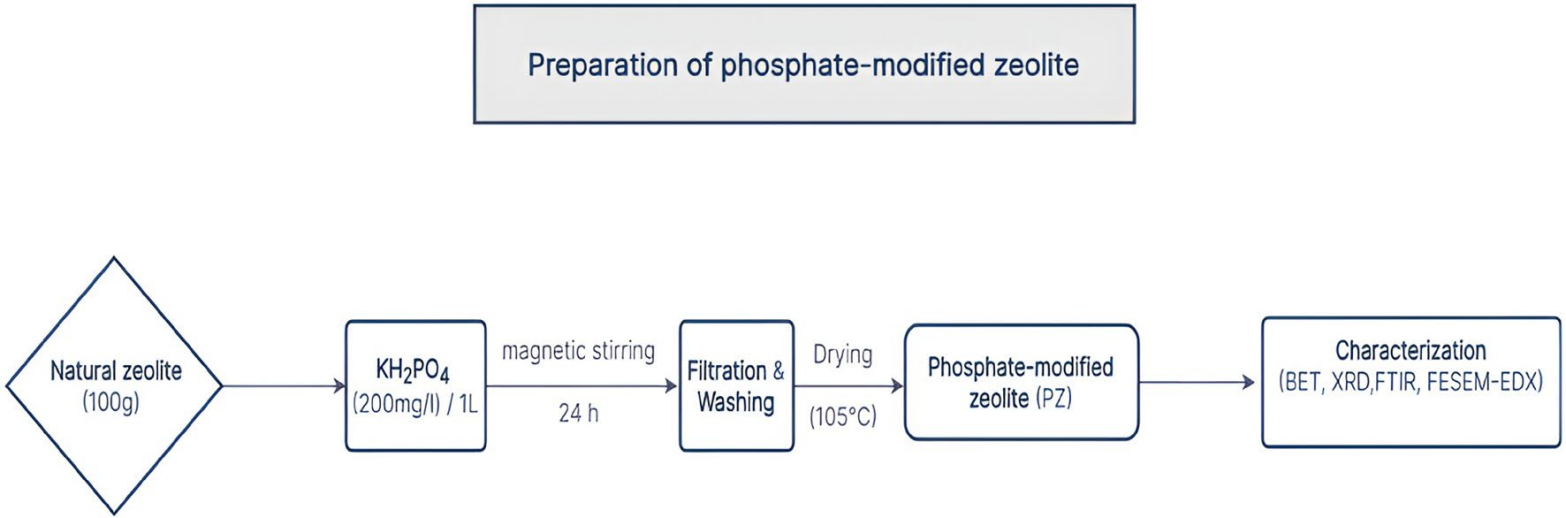
Simplified flowchart illustrating the preparation procedure of natural zeolite using phosphate anions.

Phosphate-modified zeolite was prepared by mixing 100 g of the natural zeolite (clinoptilolite) with 1000 ml of 200 mg/L of Potassium dihydrogen phosphate (KH_2_PO_4_) in 2000 ml beaker. The zeolite suspension was stirred on a magnetic stirrer (Model: Cimarec 1, Thermolyne Barnstead, USA) for 1 day at room temperature (25 °C), after which it was been filtered off by using Whatman filter papers. The solid phase was washed several times with 1000 ml portion of deionized water in order to remove excess phosphate ions. Test for phosphate in the filtered solution was confirmed negative. The phosphate-modified zeolite (PZ) was subsequently dried in an oven at 105 °C, packed into glass containers, and stored in the desiccator at room temperature (25 °C) for further use. The preparation procedure of natural zeolite using phosphate anions is illustrated in Fig. 1.

### Characterization studies

The phosphate-modified zeolite (PZ) was characterized using X-ray diffraction (XRD) to confirm the adsorbent material's identity, purity, and crystallinity. The X-ray diffraction (XRD) data of the PZ were collected at room temperature (25 °C) using a Bruker AXS Advance D8 diffractometer equipped with a monochromatic monochromatic Cu Kα radiation source (wavelength (λ) = 1.5406 Å), soller slits (0.02 rad), a fixed divergence slit (0.3°) and operated at a tube current of 40 mA and a tube voltage of 40 kV with a scanning speed of 2°/min. A Scintillation counter detector was used to scan the samples between 2θ = 5° and 50°.

The nitrogen adsorption/desorption isotherms were measured at 77 K using a Micromeritics ASAP 2020 instrument. Before performing the BET analysis, the phosphate-modified zeolite was first degassed for 10 h under vacuum conditions at 300 °C to avoid destroying the structure of the zeolite. Nitrogen adsorption/desorption isotherms were measured over the relative pressures (P/Po^−1^) ranging from 0.01 to 0.99. The specific surface area (S_BET_) of the adsorbent material was determined using the BET method and the pore size distributions were determined by Barrett-Joyner-Halenda (BJH) method from the desorption data.

To identify the functional groups contained in the phosphate-modified zeolite (PZ), the FTIR analysis equipment was used to study Fourier transform infrared spectroscopy of the adsorbent material. This instrument is equipped with a single reflection diamond ATR crystal. The Fourier-transform infrared spectra (FTIR) of the sample were obtained in the 650–4000 cm^–1^ range.

The surface morphology of the phosphate-modified zeolite (PZ) has been studied by using the field emission scanning electron microscopy (FESEM) under the following analytical conditions: EHT = 3.00 kV, Signal A = InLens and SE2, WD = 7.5, 9, 9.2 and 9.3 mm at a magnification of 10000x. The field emission scanning electron microscopy is equipped with energy dispersive X-ray spectroscopy (EDX) that was utilized to assure the natural zeolite modification procedure and to determine the dispersion of the desired species in the phosphate-modified zeolite. Positions and concentrations of different elements were determined using X-ray elemental mapping analysis (dot mapping).

### Preparation of rare earth residue sample and digestion process

The radioactive residue that was used in the current research was acquired from rare earth extraction industry situated in Malaysia. The residue was dried at 105 °C for 3 days to make sure that the most amount of moisture content was eliminated. After that, the residue was left to cool at room temperature (25 °C) for sufficient time. Then, it was ground and sieved with a mesh size of 500 μm to obtain a homogeneous fine powder.

**Figure 2:**
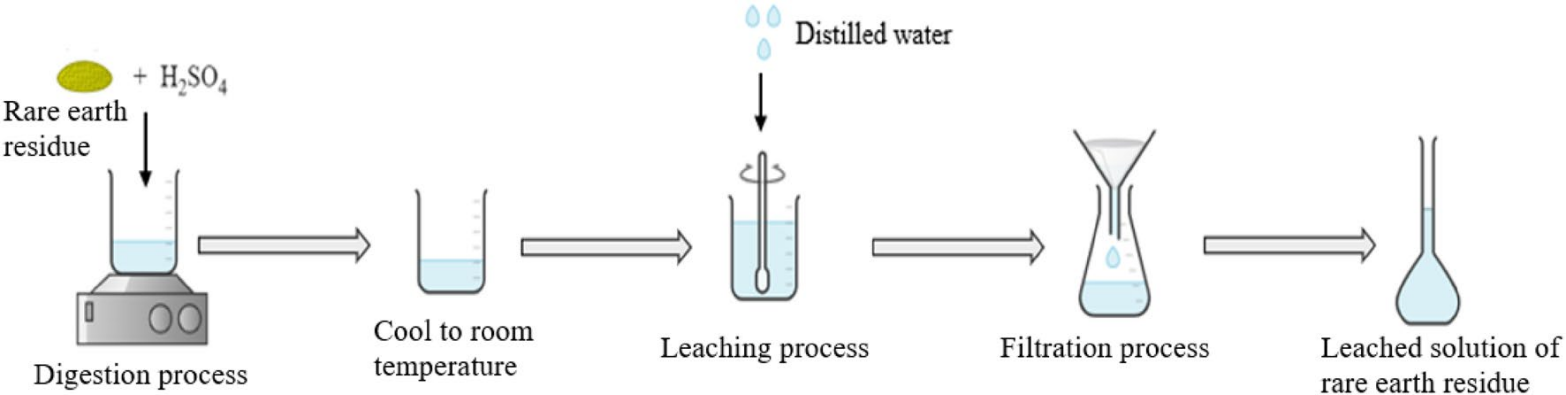
Flowchart diagram showing the digestion and leaching process of radioactive residue.

The residue was subjected to sulphuric acid leaching using optimized conditions as reported in^40^ to ensure the highest dissolution of thorium as shown in Fig. 2. The residue digestion was carried out in sulphuric acid media (18 mol/L) with a 1:5 (residue/acid) ratio. The digestion process was carried out on the stirring hot plate for two hours with constant stirring at 600 rpm at 150 °C. After 2 h, the resulting mixture was left to cool at room temperature (25 °C). A similar amount of distilled water was added to the mixture in order to dissolve the metal sulphate. After that, the leaching process takes place at room temperature for 1 h, and the solution was subsequently filtered to produce a clear leached solution of residue. The thorium and other elements concentrations in the leached solution were determined by using inductive coupled plasma-mass spectrometry (ICP-MS, Perkin Elmer Sciex Elan 9000, USA). The resulting leached solution contained 34.57 mg/L of thorium.

### Adsorption experiments

The adsorption experiments were carried out using the batch method. In the batch adsorption experiments, a suitable amount of phosphate-modified zeolite (PZ) with various concentrations of thorium solutions was shaken in 2 mL polypropylene vials for a known period of time at a constant temperature to achieve the equilibrium. The influence of the factors such as, contact time, the mass of adsorbent the initial (Th) concentration, and pH of the thorium solution (ICP-61N-5, New Haven-CT, USA) on the removal efficiency of thorium by phosphate-modified zeolite (PZ) has been investigated by changing one factor and keeping other factors constant to determine the optimum conditions. The following operating parameters were varied: contact time (from 0.25 to 30 h), adsorbent mass (from 0.005 to 0.035 g), initial thorium concentration (from 50 to 600 mg/L), and solution pH (from 2 to 6). The aqueous phase was separated by using centrifugation at 13,400 rpm for 10 min. One milliliter of the supernatant was taken from each polypropylene vial to be analyzed by using inductive coupled plasma-mass spectrometry to determine the equilibrium concentration of thorium remaining in the solutions after the adsorption processes. Besides that, the adsorption test was also carried out on the leached solution of rare earth residue using PZ.

All experiments were conducted in triplicate, and only the average results were reported. The difference between the initial and equilibrium concentration of thorium has been used to calculate the amount of the thorium adsorbed on the surface of the adsorbent (qe) by using the following Eq. (1):

1 $$qe=\frac{\left(\mathrm{Co}-\mathrm{C}e\right) V }{m},$$

where, Co is the initial concentration of the adsorbate molecules in the solution (milligram per liter), C*e* is the equilibrium concentration of adsorbate molecules remaining in the solution (milligram per liter), $V$ is the volume of solution (liter), m is the mass of absorbent material (gram), and qe is the adsorption capacity of adsorbent (milligram per gram).

The removal efficiency of the adsorbent material can be obtained using the following Eq. (2):

2 $$\mathrm{The\,\, removal \,\,efficiency} \left(\%\right)= \frac{\left(\mathrm{Co}-\mathrm{C}e\right)}{\mathrm{Co}}\times 100.$$

## Results and discussion

### Characterization of adsorbent

#### X-ray diffraction (XRD) analysis

**Figure 3:**
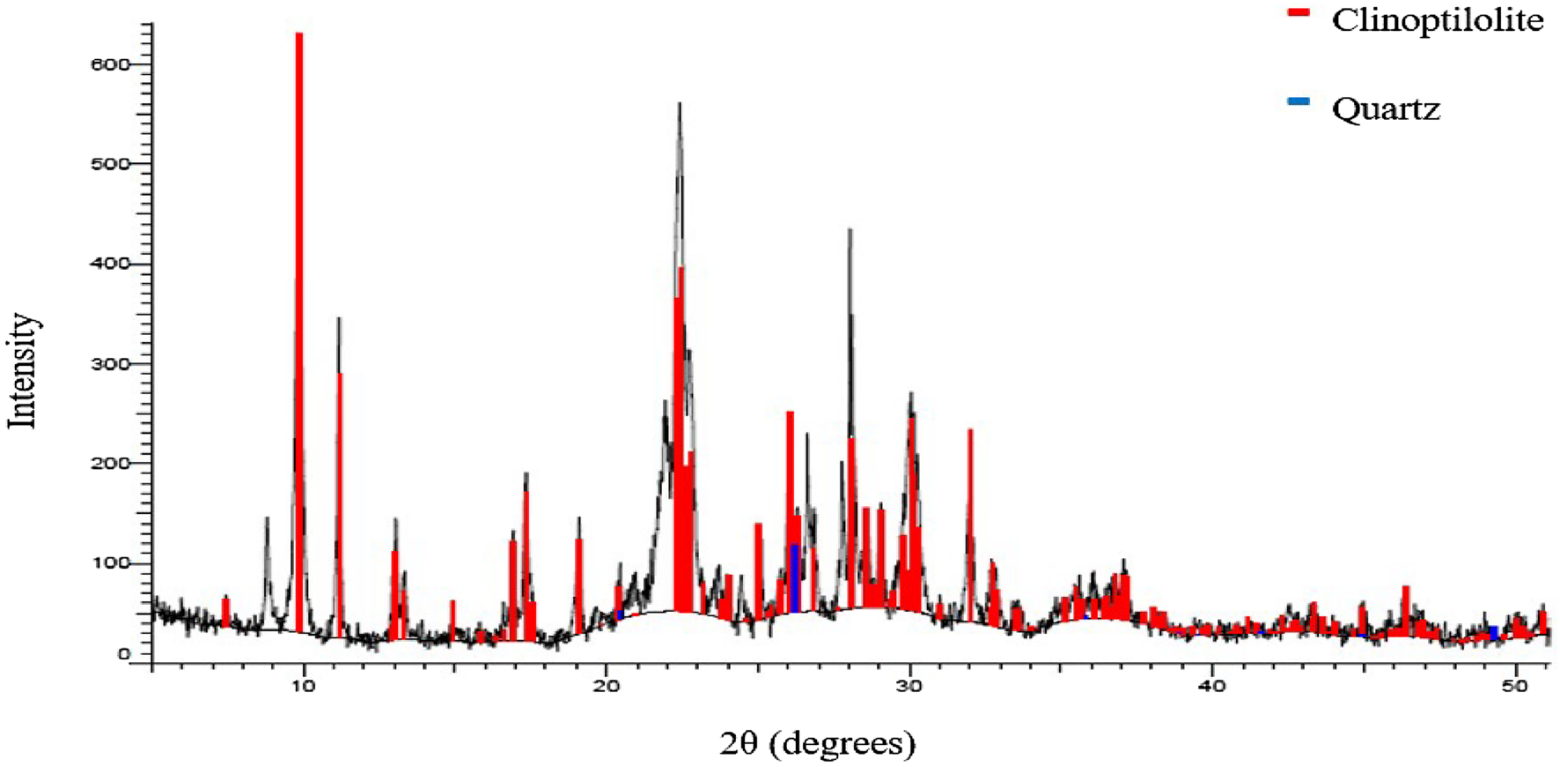
The X-ray diffraction (XRD) pattern of PZ.

Figure 3 illustrates the XRD pattern of PZ. Using Diffrac.Suite Eva software, the result of the XRD pattern was compared to the database of diffraction patterns provided by the International Center for Diffraction Database (ICDD). As can be observed, the sample contains clinoptilolite as the predominant mineral which is identified by its main distinctive peaks at 2θ = 9.87, 11.18, 13.05, 13.33, 14.93, 16.90, 17.34, 19.08, 22.48, 28.12, 30.03 and 31.99° according to (JCPDS Card No: 01-083-1261)^41^. Additionally, the XRD pattern of PZ revealed the presence of a trace quantity of quartz phase as an impurity (JCPDS Card No: 01–075-1555)^42^.

**Figure 4:**
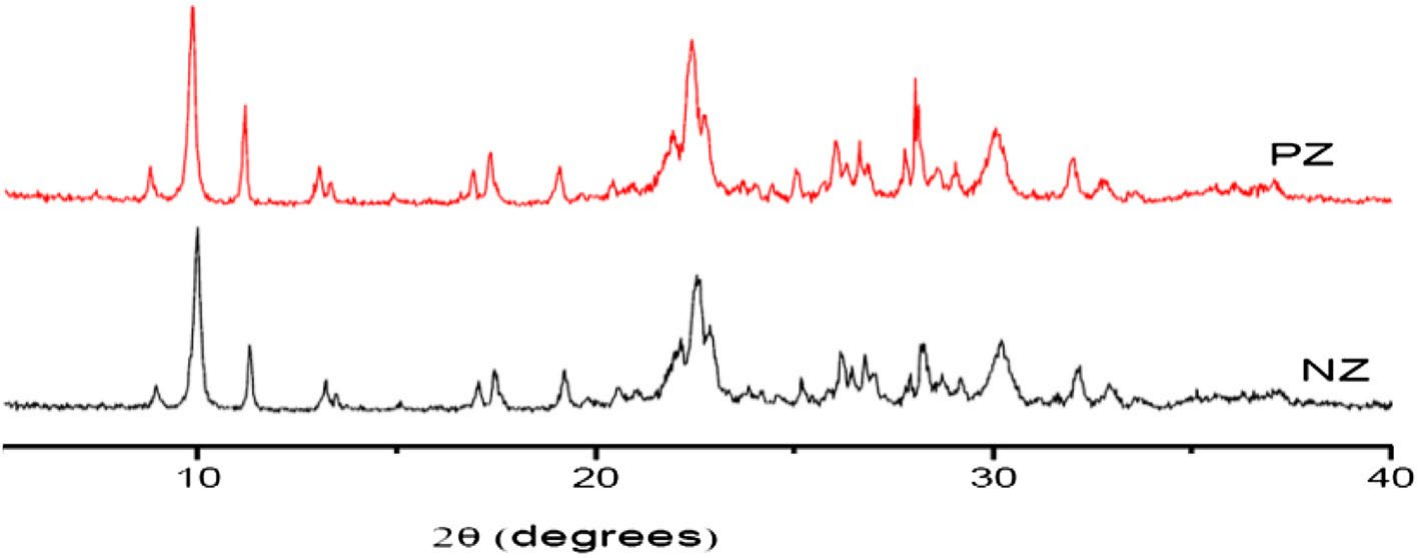
The X-ray diffraction (XRD) patterns of NZ and PZ.

Furthermore, a comparison of XRD patterns revealed no significant changes in the locations of the majority of the peaks after the modification procedure. Hence, the results demonstrated that the modification using phosphate had no effect on the main structure of the natural zeolite (NZ). However, as seen in Fig. 4, the intensity of PZ peaks increases at 2θ = 11.18, 22.48, and 28.12°. The presence of dust and other impurities in the zeolite phase is common since natural zeolite is a mineral that was extracted from deposits. As a consequence of repeatedly washing with deionized water throughout the modification process, these impurities have been eliminated from the natural zeolite structure as a result the crystallinity and peak intensities of natural zeolite are increased. The crystallinity of NZ and PZ was 82.36 and 86.38%, respectively. A comparable increase in the intensity of natural zeolite peaks was also observed by other scientists^42^.

#### N_2_ adsorption–desorption isotherm analysis

**Figure 5:**
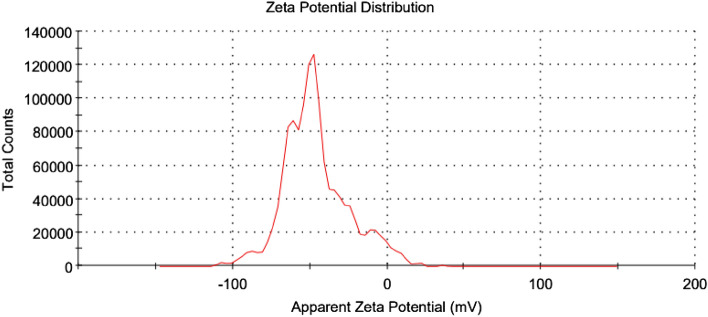
Zeta potential distribution curve of PZ.

The specific surface area (S_BET_) and other textural properties such as average pore size and total pore volume of NZ and PZ are reported in Table 1. As shown in the table, the specific surface area (S_BET_) of NZ is 28.84 m^2^/g. However, after modification of NZ using phosphate anions, the specific surface area (S_BET_) of NZ was decreased by 13.2%. The modification of NZ using phosphate anions may have resulted in pore blockage, leading to a decrease in specific surface area (S_BET_). However, this modification also introduced additional adsorption sites, thereby enhancing the adsorption capacity of the modified zeolite (PZ). The presence of phosphate anions increases the negative charge on the zeolite surface, creating extra adsorption sites that improve the maximum thorium adsorption capacity. During the modification process, phosphate anions can form bonds with bridging oxygen groups in the zeolite framework, adding two negative charges to the surface for each phosphate anion involved. Consequently, two phosphate anions may be capable of complexing the thorium ion^39^. Additionally, the zeta potential of the phosphate-modified zeolite (PZ) adsorbent was measured using a Malvern Zetasizer instrument (Malvern Instruments Ltd., United Kingdom). As depicted in Fig. 5, the PZ adsorbent exhibited a negative zeta potential of − 46.50 mV, making it an excellent material for adsorbing positively charged species like Th(IV).

**Table 1:** The textural properties of NZ and PZ.

Adsorbent materials	BET surface area (m^2^/g)	Average pore size (nm)	Total pore volume (cm^3^/g)	Reference
Natural zeolite (NZ)	28.84 ± 0.49	17.47	0.114	Present study
Phosphate modified zeolite (PZ)	25.04 ± 0.58	19.11	0.116	
Pristine bentonite	80	–	–	^43^
Modified bentonite	79	–	–	
Pristine goethite	51.18	–	–	^44^
Modified goethite	36.96	–	–

The decrease in specific surface area (S_BET_) has already been observed and reported by many researchers as shown in Table 1^43,44^.

**Figure 6:**
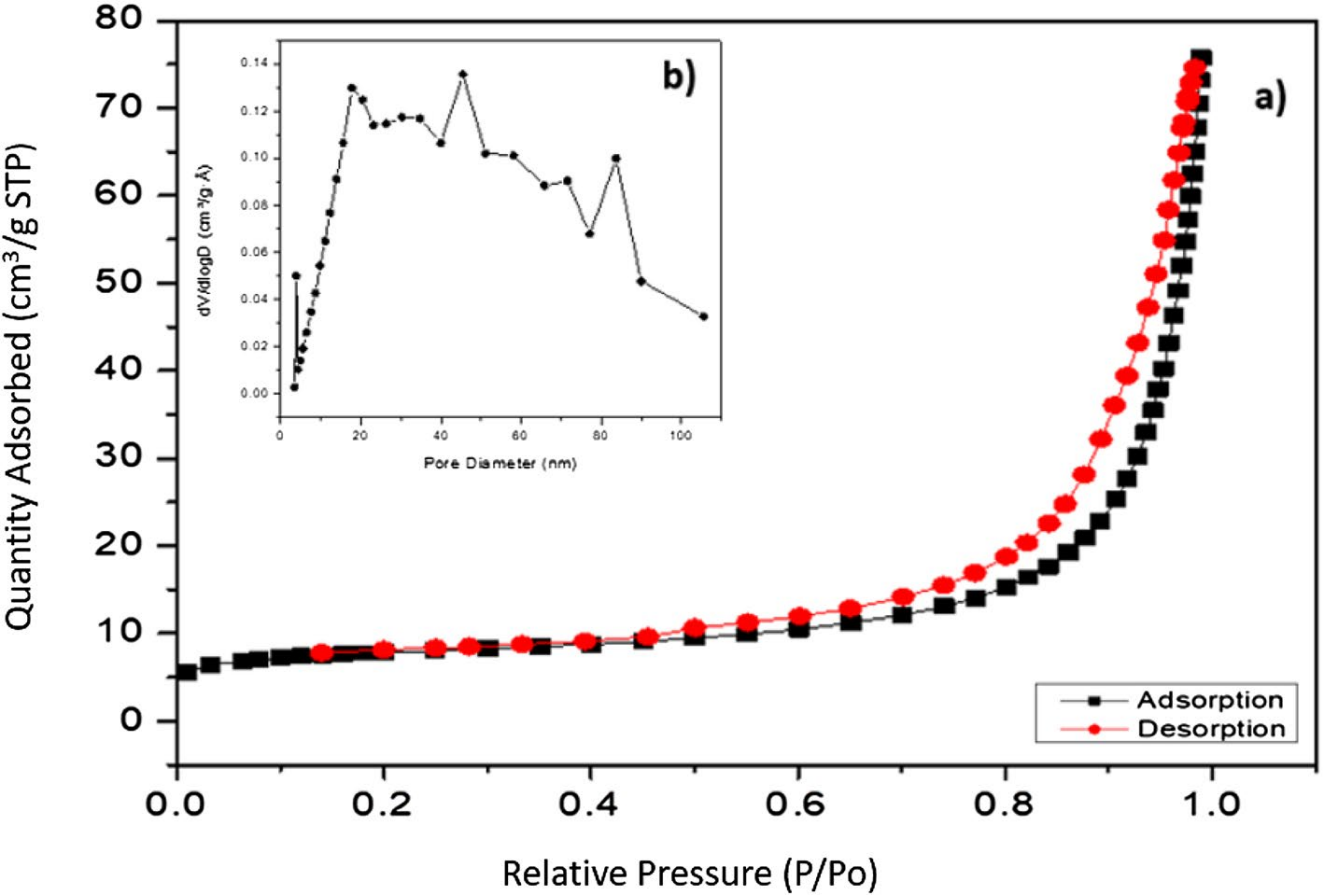
(**a**) The nitrogen adsorption/desorption isotherms of PZ; (**b**) pore size distribution of PZ.

The nitrogen adsorption/desorption isotherms of PZ are visualized in Fig. 6a. According to the International Union of Pure and Applied Chemistry (IUPAC) for porous materials, the adsorption/desorption isotherms obtained can be classified as type IV which is the characteristic of mesoporous materials. The nitrogen adsorption/desorption isotherms of PZ are accompanied by a type H3 of the hysteresis loop, in the range 0.4 – 0.99 P/Po. The presence of a hysteresis loop of this type is considered to be a clinoptilolite material characteristic, which can be attributed to multilayer physical adsorption followed by capillary condensation either in the space between the crystallites of zeolite or in mesopores of impurities (quartz, feldspars, etc.)^45,46^.

Figure 6b displays the pore size distribution of PZ, derived from the desorption data of the nitrogen adsorption/desorption isotherms using the BJH method. According to the IUPAC classification, pores can be divided into three main categories: micropores (diameter < 2 nm), mesopores (2–50 nm), and macropores (diameter > 50 nm). As shown in Fig. 6b, the analysis of pore size suggests that PZ is mesoporous material, as most of its pore diameters are in the range of 2–50 nm.

#### Fourier transforms infra-red (FTIR) analysis

**Figure 7:**
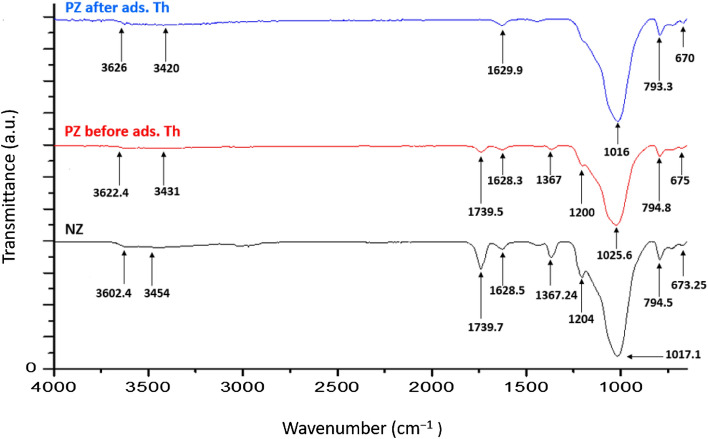
The FTIR spectrum of (NZ) and (PZ before and after the adsorption of thorium).

Figure 7 shows the FTIR spectrum of NZ and PZ. Based on the spectrum of NZ, the existing functional groups are as follows: T–O (T=Si or Al) in wavenumber 1017.1 cm^–1^, (Si, Al)O_4_ in 1203.43 cm^–1^, H–O–H in the wavenumber 1628.5 cm^–1^ which corresponded to harmonic vibration, and –OH group in the wavenumber 3454 cm^–1^ which corresponded to stretching vibration. The peaks identified on the NZ spectra are similar to the infrared testing results of some zeolites^47,48^.

Modification of natural zeolite using phosphate anions caused a shift in the structural –OH vibrational band from 3602.4 and 3454 cm^–1^ to 3622.4 and 3431 cm^–1^, respectively. The stretching and bending vibrations in NZ shifted from 1017.1 to 1025.6 cm^−1^. The 1025.6 is the P–O stretching peak. This stretching vibration of P–O group is usually observed between 1000 and 1120 cm^−1^^49^. This peak usually appears as a strong band as shown in Fig. 7. According to the analysis of the previous FTIR spectra, it is therefore highly likely to conclude that the modification of natural zeolite using phosphate was effective on the natural zeolite surface. After the adsorption of thorium ions on PZ, the peaks at 1200, 1367, and 1739.5 cm^-1^ almost disappeared. The majority of bands were shifted, confirming the adsorption of thorium onto PZ adsorbent as presented in Fig. 7. According to study^50^, FTIR analysis also showed a similar pattern in which peaks disappeared and shifted after thorium adsorption.

#### Field emission scanning electron microscopy (FESEM) and EDX analysis

**Figure 8:**
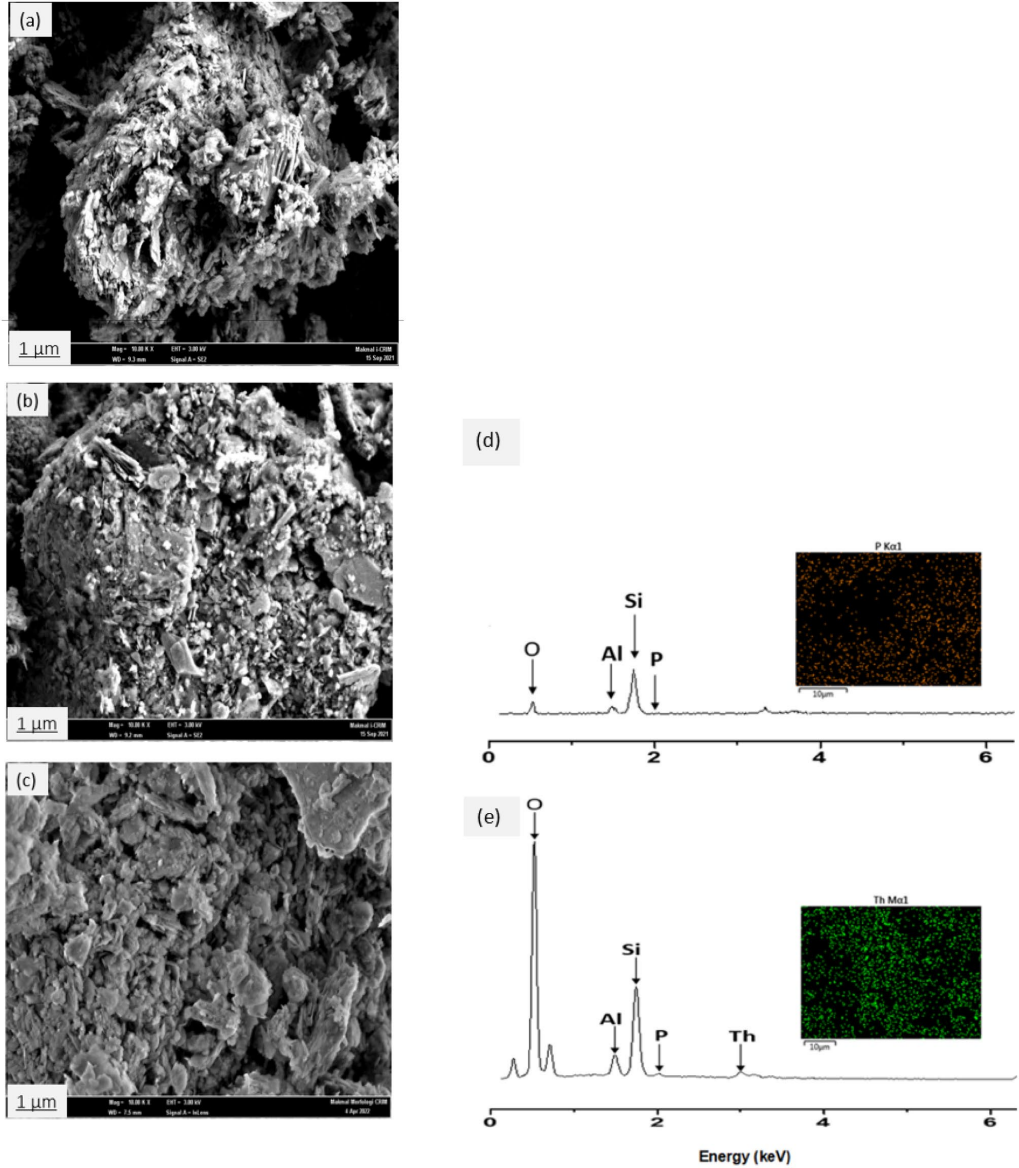
FESEM and EDX spectrum analysis, the FESEM image of (**a**) NZ and PZ (**b**) before and (**c**) after the adsorption of thorium, at a magnification of 10000x; the EDX spectrum and dot mapping of PZ (**d**) and PZ adsorbed thorium (**e**).

In this study, the surface morphology of natural zeolite (NZ) and phosphate-modified zeolite (PZ) before and after thorium adsorption was investigated using field emission scanning electron microscopy (FESEM) equipped with energy dispersive X-ray spectroscopy (EDX). Figure 8a and b present the surface morphology of NZ and PZ, respectively. The FESEM images revealed that the crystals in both samples appeared as plates and laths, which is consistent with previous findings^51^. The micrographs indicated that there were no significant changes in the surface morphology of NZ after the modification process, and the particle and pore sizes of PZ were similar to those of NZ. Figure 8c shows the surface morphology of PZ after thorium adsorption.

Figure 8d and e present the EDX spectrum and mapping of PZ before and after thorium adsorption. The results of the EDX analysis reveal the presence of the P element in the phosphate-modified zeolite structure, as depicted in Fig. 8d, which is consistent with the FTIR analysis. These results provide strong evidence of the successful modification of the natural zeolite surface. The adsorption of thorium on PZ is confirmed by both EDX analysis and elemental mapping, as presented in Fig. 8e. The elemental mapping shows the presence of thorium at approximately 3 keV, confirming the adsorption of thorium onto the surface of PZ.

### Effect of various experimental conditions on thorium adsorption

#### Effect of contact time

**Figure 9:**
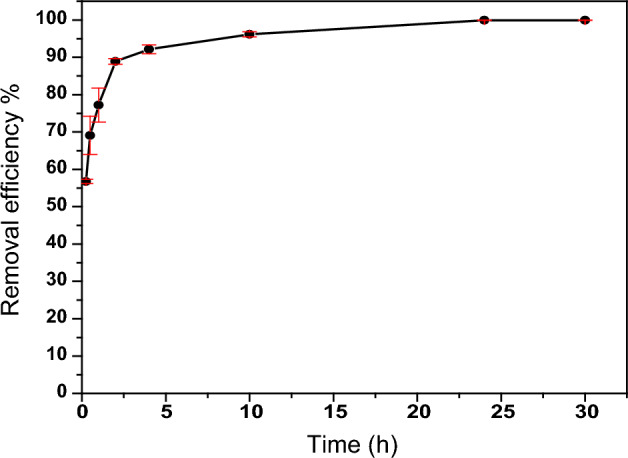
Effect of contact time on the removal efficiency of Th by PZ adsorbent. Adsorption conditions: (100 mg/L) Th, pH (3), room temperature, (2 mL) of thorium solution, and (0.03 g) of PZ.

In this study, the impact of contact time on the effectiveness of PZ adsorbent in removing thorium ions was investigated. The contact time was modified from 0.25 to 30 h, while keeping other conditions constant. Figure 9 illustrates the relationship between contact time and thorium removal efficiency by PZ. The results reveal that the removal efficiency of thorium increased rapidly with an increase in contact time, reaching 56.7% at 0.25 h and 88.9% at 2 h. Equilibrium was established at 24 h with 99.9% removal efficiency, and no substantial increase was observed beyond this point. Thus, 24 h was selected for the subsequent experiments to ensure equilibrium was attained.

The adsorption process is typically divided into three stages: (1) the first stage involving rapid adsorption, (2) a subsequent slower stage, and (3) the third stage at which adsorption reaches equilibrium and remains constant. Rapid adsorption in the first stage is attributed to the rapid attachment of adsorbate molecules to the adsorbent surface through surface mass transfer. The second stage is slower, possibly due to many available adsorption sites in the first stage already being occupied. In the last stage, available adsorption sites on the adsorbent surface become rare, leading to the equilibrium state^52^. For example, clinoptilolite was used to remove thorium ions from aqueous solutions in^37^, where a rapid adsorption rate was observed in the first two hours, followed by a slow adsorption process until equilibrium was reached after 24 h.

#### Effect of adsorbent mass

The amount of adsorbent mass used is a critical factor in determining the removal efficiency of adsorbate molecules from the solution. As the adsorbent mass increases, the contact area between the adsorbate molecules and the adsorbent also increases. Therefore, it is necessary to find the optimal adsorbent mass to achieve maximum removal efficiency while minimizing the use of the adsorbent to maintain cost-effectiveness. In this research, a series of experiments were conducted to examine the impact of PZ adsorbent mass on the elimination of thorium ions at seven different masses ranging from 0.005 to 0.035 g of PZ, all other conditions were kept constant.

**Figure 10:**
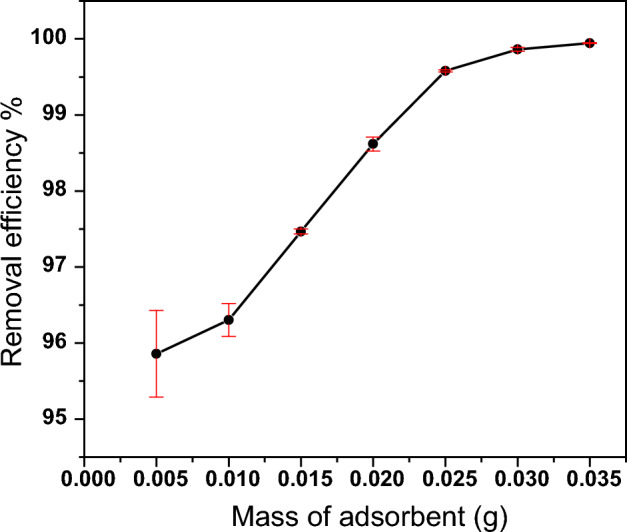
Effect of PZ adsorbent mass on the removal efficiency of Th. Adsorption conditions: (100 mg/L) Th, pH (3), room temperature, (24 h) contact time and (2 mL) of thorium solution.

Figure 10 depicts the relationship between the PZ adsorbent mass and the percentage of thorium removal from the solution. The findings revealed that an increase in the adsorbent mass from 0.005 to 0.03 g resulted in an increase in the removal of thorium ions from 95.85 to 99.86%. This enhancement in removal efficiency was likely due to the greater number of adsorption sites provided by the PZ adsorbent in the solution. However, the removal efficiency did not significantly increase beyond 0.03 g mass, which can be attributed to the overlapping or aggregation of adsorbent particles^53^. Previous studies have also reported similar trends^22,54^. Therefore, to optimize the use of absorbent material, 0.03 g of PZ adsorbent was selected as the optimum mass for subsequent experiments.

#### Effect of the initial concentration of thorium

One of the most important factors in the adsorption system, which can influence the behavior of adsorption, is the initial concentration of the metal ions. The percentage of thorium ions removed is highly dependent on the initial thorium concentration. The effect of the initial concentration factor relies on the direct relationship between the initial thorium concentration in the solution and the available adsorption sites on the surface of the adsorbent. Therefore, the effect of the initial thorium concentration on the adsorption process was investigated.

**Figure 11:**
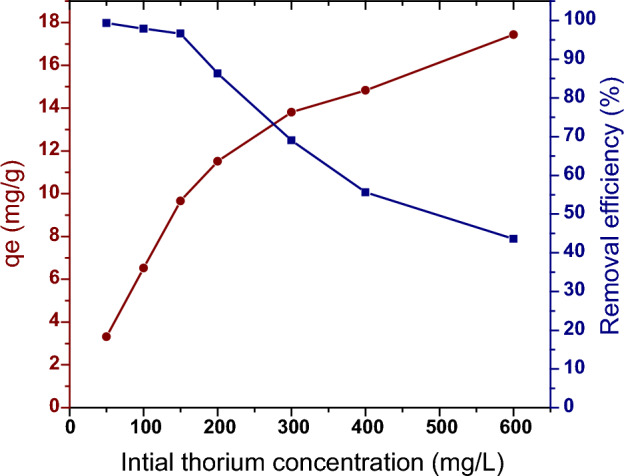
Effect of initial thorium concentration on the adsorption of Th onto PZ adsorbent. Adsorption conditions: pH (3), room temperature, (24 h) contact time, (2 mL) of thorium solution and (0.03 g) of PZ.

The impact of the initial concentration of thorium on the adsorption process using PZ adsorbent was investigated, as it is a significant factor that can influence the adsorption behavior. Figure 11 displays the effect of the initial concentration of thorium on its adsorption onto PZ adsorbent by increasing its initial concentration from 50 to 600 mg/L, while keeping other conditions constant. The results indicate that the thorium removal percentage decreased from 99.32 to 43.58% with an increase in the initial thorium concentration from 50 to 600 mg/L. This is because the PZ surface became saturated. At low thorium concentrations, unoccupied adsorption sites existed on the PZ surface, but as the initial concentration increased, the adsorption sites became occupied and disappeared^55^. However, the adsorption capacity (qe) increased from 3.31 to 17.43 mg/g, which might be attributed to an increase in the driving force for mass transfer of the metal ions from the bulk solution to adsorption sites as the initial concentration increased^55,56^. Similar results have been reported for the adsorption of thorium^37,54^.

#### Effect of pH

The adsorption of metal ions is strongly influenced by the pH of the aqueous solution, which can alter the surface properties and charge of the adsorbent material, as well as the speciation and precipitation of the adsorbate molecule^57,58^. In this study, the impact of pH on the adsorption of thorium onto Phosphate-modified zeolite (PZ) was examined over a pH range of 2–6, while keeping all other conditions constant.

**Figure 12:**
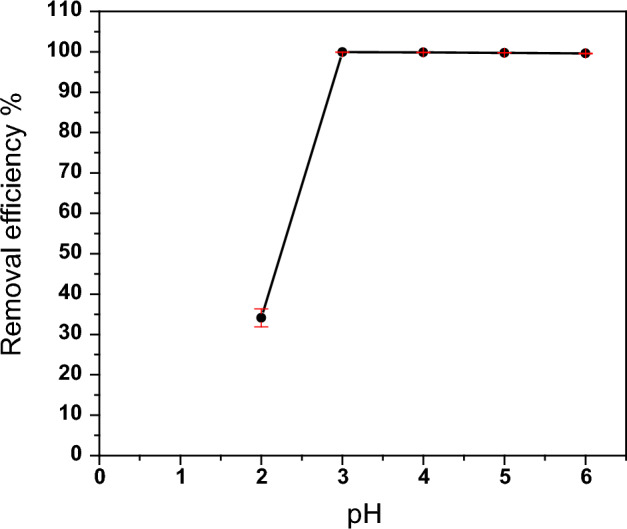
Effect of pH on the adsorption of Th onto PZ adsorbent. Adsorption conditions: (100 mg/L) Th, (24 h) contact time, room temperature, (2 ml) of thorium solution, and (0.03 g) of PZ.

The impact of pH on thorium adsorption onto the PZ adsorbent is presented in Fig. 12. The adsorption process can be divided into two distinct phases based on the figure. The first phase is characterized by a rapid increase in thorium removal efficiency from 34.11 to 99.91% with the increase in pH from 2 to 3. In the second phase, thorium removal efficiency remains high even as pH changes from 4 to 6. At pH levels lower than 3, a decrease in thorium removal efficiency is observed due to competition between Th(IV) ions and H^+^ (or H_3_O^+^) ions for adsorption sites on the PZ adsorbent surface^59–61^. Raising the pH value from 2 to 3 reduces the number of H^+^ (or H_3_O^+^) ions, leading to increased thorium ion adsorption from the solution and improved removal efficiency that reaches a maximum at pH 3, indicating that the process occurs via adsorption^62,63^.

It should be noted that the distribution of thorium ion species changes with variations in the pH of the solution. At pH ≤ 3, Th(IV) is the dominant species with over 88%, while Th(OH)^3+^ accounts for the remaining species^64,65^. However, at pH values higher than 4, the role of precipitation and hydrolysis products becomes significant in Th(IV) adsorption. Hence, the thorium removal efficiency remains high as the pH value increases from 4 to 6^63,66^. To prevent any precipitation during the adsorption process, a pH of 3 was selected as the optimal condition for conducting the subsequent adsorption experiments.

### Comparison of the adsorption efficiency of PZ with natural zeolite

To demonstrate the potential benefits of using modified zeolite for thorium removal, the optimal adsorption conditions (0.03 g of adsorbent, 50–600 mg/L thorium ion, 2 ml of solution, pH 3, shaking for 24 h at room temperature) were evaluated using natural zeolite (clinoptilolite) as an unmodified control. Results indicated that the thorium removal efficiency decreased significantly from 99 to 25% when using unmodified zeolite.

### Adsorption isotherms

Adsorption isotherm models are commonly used to describe the relationship between the amount of adsorbate molecules adsorbed and the equilibrium concentration of adsorbate remaining in solution at a constant temperature. These models are useful for understanding the adsorption mechanism, capacity, and force. There are many different adsorption isotherm models available, but in this study, the Langmuir, Freundlich, and Dubinin-Radushkevitch (D-R) isotherm models were selected to analyze the equilibrium data of adsorption**.**

To determine the best-fitting adsorption isotherm model, experiments were conducted using various initial concentrations of thorium solutions (ranging from 50 to 600 mg/L), with 2 ml of solution, 0.03 g of PZ, pH 3, at room temperature (25 °C), and shaking at 775 rpm for 24 h.

#### Langmuir adsorption isotherm model

**Figure 13:**
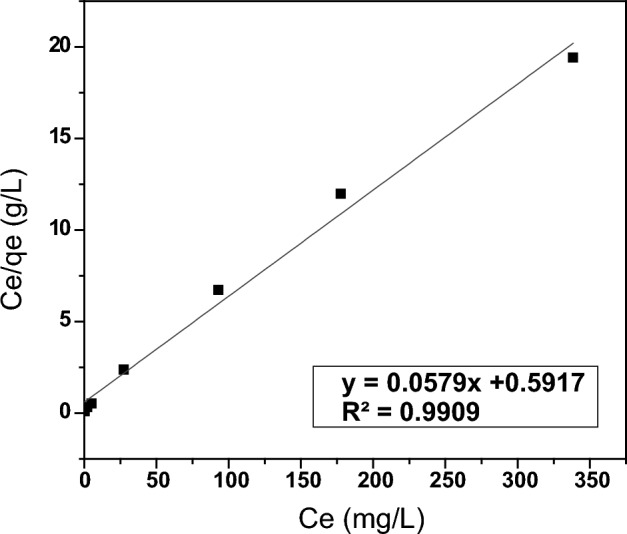
Langmuir isotherm model of thorium adsorption onto PZ.

The Langmuir adsorption isotherm model posits that adsorption occurs on a uniform surface with identical adsorption sites. Additionally, when adsorption reaches its limit, a monolayer of adsorbate molecules forms on the surface of the adsorbent, with no interaction between them. Equation (3) expresses the linearized form of the Langmuir isotherm model^67^:

3 $$\frac{\mathrm{C}e}{qe}=\frac{1}{\mathrm{Qo }\times {\mathrm{K}}_{\mathrm{L}}}+\left[\frac{1}{\mathrm{Qo }}\right]\mathrm{C}e,$$

where C*e* is the equilibrium concentration of thorium ions in the solution (mg/L), *qe* is the amount of adsorbed thorium ions per PZ unit weight at equilibrium state (mg/g), K_L_ is the Langmuir isotherm coefficient (L/mg) and Qo is the maximum adsorption capacity of thorium ions (mg/g). A plot of Ce/qe versus Ce yields a straight line of which the slope is equal to (1/Qo), and the intercept (1/Qo × K_L_) as shown in Fig. 13. The following outcomes were acquired: Qo = 17.3 mg/g, which agrees well with the experimental capacity, K_L_ = 0.09 L/mg, and correlation coefficient R^2^ = 0.99 (regression equation y = 0.0579x + 0.5917). The outcomes indicate that the use of the Langmuir adsorption isotherm model for describing the adsorption process of thorium ions on the investigated adsorbent PZ is valid. The Langmuir isotherm model's accessibility indicates that there is no interaction between the adsorbate molecules and that the adsorption of thorium ions onto the studied adsorbent takes place at homogeneous adsorption sites on the surface of the adsorbent to form one monolayer.

The dimensionless separation factor (R_L_) is the essential feature of the Langmuir isothermal modeling. It could be utilized to predict whether an adsorption process is favorable or unfavorable. R_L_ can be defined by the following Eq. (4)^68^:

4 $${R}_{L}=\frac{1}{(1+{K}_{L} \times {C}_{o})},$$

where K_L_ is the Langmuir isotherm coefficient (L/mg), and C_o_ is the initial thorium concentration in the solution (mg/L). The adsorption process is favorable at (0 < R_L_ < 1), unfavorable at (R_L_ > 1), linear at (R_L_ = 1) and irreversible at (R_L_ = 0). The calculated R_L_ values for thorium adsorption onto the studied adsorbent PZ were found to be 0 < R_L_ < 1 in all cases, this indicates that the adsorption of thorium onto PZ is favorable under the conditions used in this research.

#### Freundlich adsorption isotherm model

**Figure 14:**
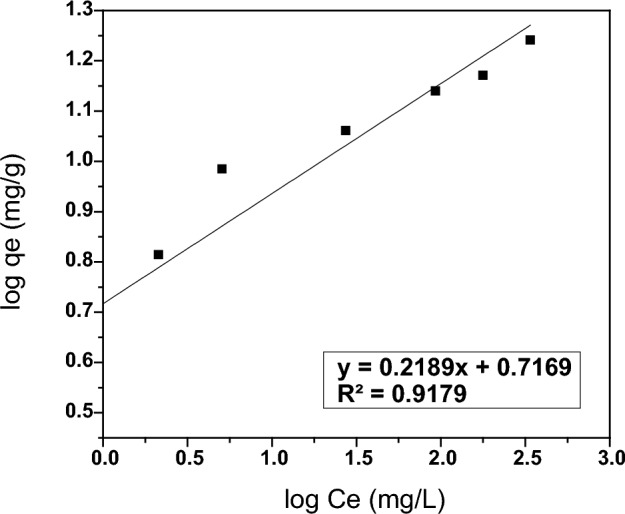
Freundlich isotherm model of thorium adsorption onto PZ.

The Freundlich adsorption isotherm model assumes that the adsorption process takes place on heterogeneous surfaces and involves multilayer adsorption with interactions between adsorbate molecules on the adsorbent’s surface. The linearized equation of the Freundlich isotherm model can be represented by Eq. (5)^69^:

5 $$\mathrm{log }\,qe =\mathrm{log }\,{\mathrm{K}}_{\mathrm{f}}+ \left(\frac{1}{\mathrm{n}}\right)\mathrm{log \,C}e,$$

where *qe* is the amount of adsorbed thorium at equilibrium state (mg/g), C*e* is the thorium equilibrium concentration in the solution (mg/L), K_f_ (mg/g), and n (unitless) are the Freundlich constant, which is indicative of the adsorption capacity of thorium and the intensity of the adsorption process, respectively. The Freundlich constants (K_f_ and n) were calculated from the slope and intercept of the linear plot of log *qe* versus log C*e* as shown in Fig. 14. The adsorption parameters acquired are as follows: K_f_ = 5.21 mg/g, n = 4.56 and the regression equation is y = 0.2189x + 0.7169 (R^2^ = 0.91). the K_f_ value is lower than the experimental data of PZ for thorium adsorption at room temperature. Consequently, the Freundlich isotherm model does not fit the thorium adsorption onto PZ.

#### Dubinin-Radushkevich isotherm (D-R)

**Figure 15:**
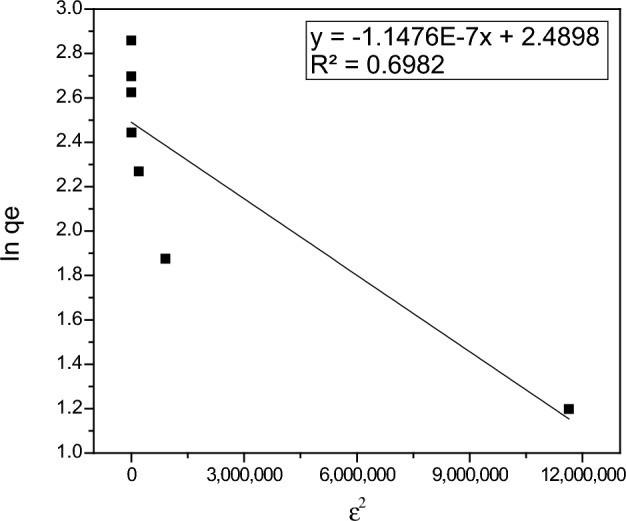
Dubinin-Radushkevitch (D-R) isotherm model of thorium adsorption onto PZ.

The Dubinin-Radushkevitch (D-R) model is more generic than the Langmuir model because it does not assume a homogeneous surface or a constant adsorption potential. The D-R model can be used to explain adsorption on homogeneous and heterogeneous surfaces^70,71^. The linearized form of the D–R model is given by the following Eq. (6)^72^:

6 $$\mathrm{In} \,qe=\mathrm{In }\,{\mathrm{q}}_{\mathrm{max}}-K {\varepsilon }^{2},$$

where *qe* is the amount of adsorbed thorium ions at equilibrium state (mg/g), q_max_ is the maximum adsorption capacity of thorium ions (mg/g), K is a constant that relates to the adsorption energy (mol^2^/kJ^2^), and ε is Polanyi potential (kJ/mol), which can be expressed as the following Eq. (7):

7 $$\upvarepsilon =\mathrm{ RT\, In}\left(1+\frac{1}{\mathrm{C}e}\right),$$

where R is the gas constant (8.314 kJ/mol.K), T is the temperature (K) and C*e* is the equilibrium concentration of thorium ions in the solution (mg/L). A plot of In *qe* versus ε^2^ yields a linear relationship of which the slope is equal to K, and intercept (In q_max_) as shown in Fig. 15. The following results were obtained: q_max_ = 12.05 mg/g, K = 1.15 × 10^–7^ mol^2^/kJ^2^, and the regression equation is y = − 1.1476 × 10^-7^x + 2.4898. The D–R model inappropriately describes the thorium adsorption onto PZ since the correlation coefficient R^2^ value is 0.69.

Table 2 compares the thorium adsorption capacities of different adsorbents in aqueous solutions under various experimental conditions. The maximum adsorption capacity of thorium onto modified zeolite (PZ) is clearly better than that of the majority of other stated adsorbents, as shown in this table. These results suggest that modified zeolite (PZ) could be a promising alternative adsorbent for removing thorium from aqueous solutions.

**Table 2:** The maximum adsorption capacity of thorium onto different adsorbents.

Adsorbent	Qo (mg/g)	Contact time (min)	Adsorbent mass (g/L)	pH	Temperature (°C)	Reference
Synthetic zeolite	2.82	2880	0.5	3.6	25	^73^
Oxidized multi-wall carbon nanotubes (MWCNTs)	13.22	1440	0.25	1.9	30	^74^
1-(2-pyridylazo)-2-naphthol (PAN)/zeolite	9.28	45	3	4	25	^75^
Merrifield polymer-DMDBMA	15.74	45	20	–	25	^76^
Na-bentonite	11.4	2880	0.1	3.5	75	^77^
Illite	7.169	30	5	3	44	^24^
Monomodified b-cyclodextrin polyrotaxane	12.92	35	1	4	25	^78^
Activated bentonite	14.3	1440	0.3	2.5	45	^21^
Acrylic fber waste/sargassum (AFWS)	59.4	60	5	3	25	^79^
Zeolite	0.24	120	–	3	–	^20^
Mesoporous Al_2_O_3_	11.72	60	5	1	55	^80^
Functionalized calix[4]arenes with n-propyl	12.4	60	–	–	25	^81^
Modified zeolite (PZ)	17.3	1440	15	3	25	Present work

### Adsorption kinetics

The present study applied four widely used adsorption kinetic models, the pseudo-first-order model, pseudo-second-order model, Elovich kinetic model and Intraparticle diffusion model to evaluate the adsorption rate and possibly determine the adsorption mechanism of thorium ions onto PZ adsorbent.

The linear form of the pseudo-first-order model equation can be expressed as the following equation^82^:

8 $$\mathrm{log}\left({q}_{e}-{q}_{t}\right)=\mathrm{log}{q}_{e}-\left(\frac{{k}_{1}}{2.303}\right)t,$$

where q_e_ and q_t_ (mg/g) are the amounts of thorium ions adsorbed onto the adsorbent surface at the point of equilibrium and at time t (min), respectively, and k_1_ is the pseudo-first-order adsorption rate constant (min^−1^).

The pseudo-second-order model is represented by the following equation:

9 $$\frac{t}{{q}_{t}}=\frac{1}{{k}_{2}{q}_{e}^{2}}+\left(\frac{1}{{q}_{e}}\right)t,$$

where k_2_ is the pseudo-second-order adsorption rate constant (g/(mg.min)).

Elovich kinetic model is expressed as^83^:

10 $${q}_{t}=A+BInt,$$

where A and B are Elovich model constants.

Intraparticle diffusion model is expressed as the following equation^59^:

11 $${q}_{t}={K}_{i}{t}^{0.5}+C,$$

where k_i_ is the Intraparticle diffusion rate constant (mg g^–1^ min^–1/2^) and C is a constant that gives information about the boundary layer.

**Figure 16:**
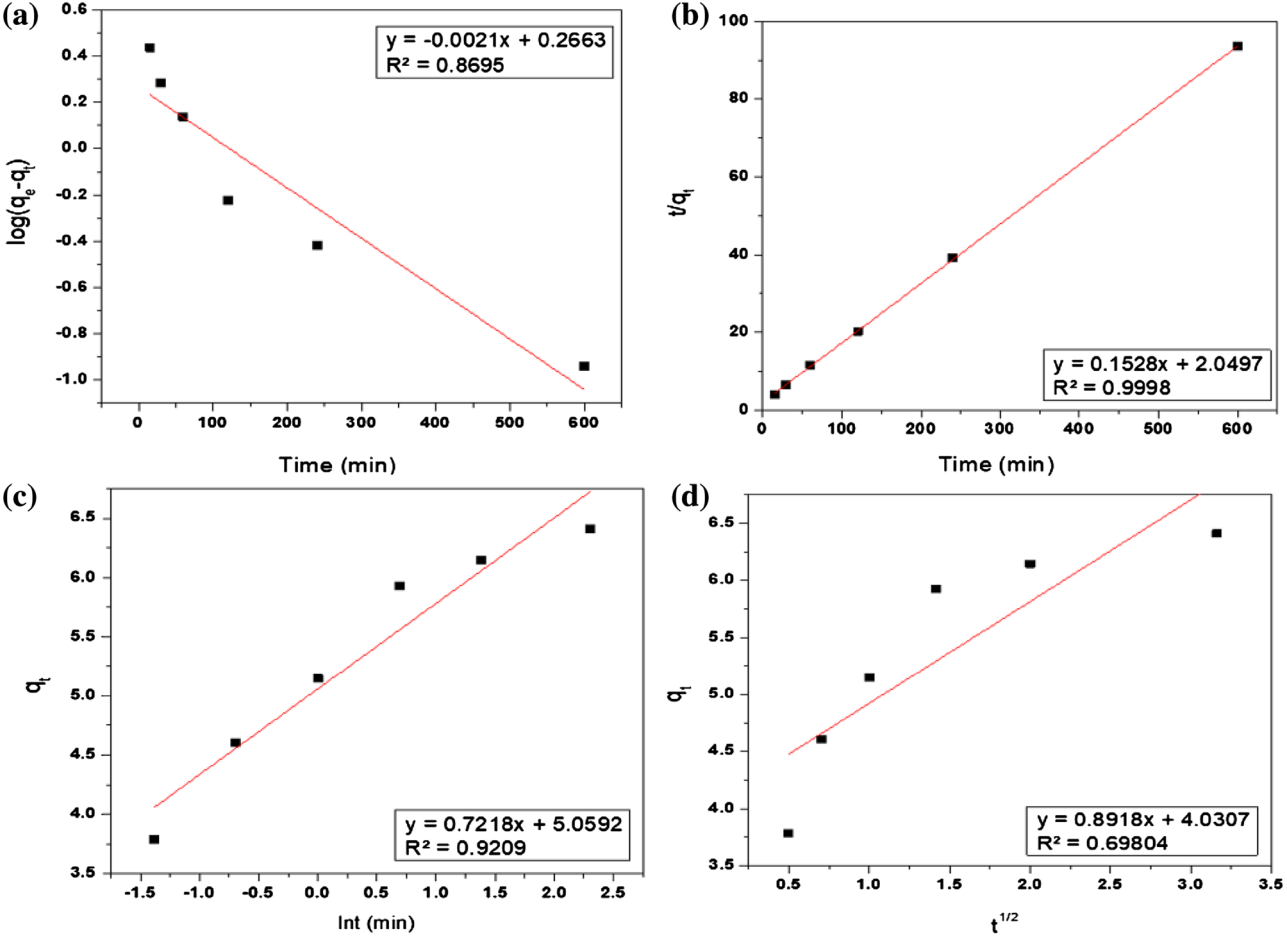
(**a**)The pseudo-first-order model, (**b**) pseudo-second-order model, (**c**) Elovich model, and (**d**) Intraparticle diffusion model for the adsorption of thorium onto PZ.

The kinetic experiments maintained fixed adsorption conditions, including a 2 ml solution of 100 mg/L thorium, pH 3, 0.03 g of PZ adsorbent, shaking at 775 rpm, and room temperature (25 °C). Figure 16 displays the pseudo-first-order model, pseudo-second-order model, Elovich kinetic model, and Intraparticle diffusion model, while Table 3 presents the calculated results of these models. According to the pseudo-second-order model, the adsorption capacity q_e_ of thorium ions onto PZ was determined to be 6.54 mg/g, which closely matched the experimental data of 6.52 mg/g. Additionally, this model exhibited a high correlation coefficient (R^2^ = 0.9998), indicating a superior fit compared to the other models for thorium adsorption. Consequently, the adsorption of thorium onto PZ is primarily governed by chemisorption, involving a chemical bond between the active sites of the adsorbent and thorium ions^14,84^. This finding aligns with previous studies on the adsorption of thorium ions using modified bentonite^54^, chitosan^23^ and silica monoliths^33^.

**Table 3:** Adsorption kinetic parameters and the correlation coefficient values (R^2^) for the adsorption of thorium on PZ.

Pseudo-first-order model			Pseudo-second-order model		
k_1_ (min^−1^)	q_e_ (mg/g)	R^2^	k_2_ (g/(mg.min))	q_e_ (mg/g)	R^2^
0.00502	1.84	0.8695	0.0114	6.54	0.9998

Elovich model			Intraparticle diffusion model		
A	B	R^2^	C	k_i_	R^2^
5.0592	0.7218	0.9209	4.0307	0.8918	0.69804

### Application of adsorption process to remove thorium from radioactive waste

The 500 mesh particle size radioactive residue underwent cracking and leaching with sulphuric acid under the specified conditions. Apart from thorium, the resulting leached solution from residue contained several other metal ions, and their concentrations were measured and reported in Table 3. Analysis of the data reveals that cerium is the most abundant element in the leached solution, with a concentration 8.4 times higher than that of thorium. Table 4. The thorium and other elements concentrations in the leached solution determined by ICP-MS.

**Table 4:** The thorium and other elements concentrations in the leached solution determined by ICP-MS.

Element	Concentration (mg/L)	Ratio metal ion to thorium (M: Th)
Ce	293.74	8.4:1
La	121.93	3.5:1
Nd	123.74	3.5:1
Pr	35.11	1.01:1
Sm	13.92	0.4:1
Eu	2.62	0.07:1
Gd	6.66	0.19:1
Tb	0.58	0.01
Dy	1.41	0.04:1
Th	34.56	–

**Figure 17:**
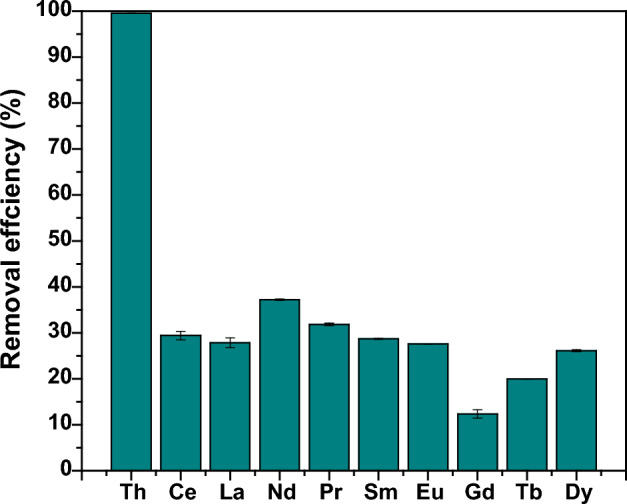
The removal efficiency of thorium and the other metal ions from the leached solution.

Then, 2 ml of this leached solution adjusted to pH 3 was shaken at 775 rpm with 0.03 g of PZ adsorbent for 24 h at room temperature (25 °C). The adsorption process on leached solution was fixed at pH 3 due to the adsorption of thorium on PZ in this study, which was in optimum condition. In addition to that, the chosen pH for the leached solution was consistent with the study by Yousef et al.^50^ where the optimum adsorption of thorium from the sulphate medium of leached solution of monazite was achieved around pH 3^50^. The concentration of elements in the leached solution of residue before and after the adsorption process determined by ICP-MS and given in Table 5. As can be seen that the concentration of thorium in the leached solution after the adsorption process was found to be 0.15 mg/L, which corresponds to a removal efficiency of 99.55%. Figure 17 shows the performance of the modified zeolite PZ adsorbent in the removal of thorium from the leached solution with the existence of the competing ions such as cerium, lanthanum, neodymium, and praseodymium. The results obtained showed that PZ adsorbent is highly selective to thorium. This might be because thorium ions have a stronger electrostatic force toward the adsorbent surface than the other competing ions^83,85–87^. These findings demonstrate that PZ can be used successfully as an adsorbent in the treatment of aqueous radioactive waste containing thorium and that it can remove more than 99% of the thorium ion even when other competing ions are present.

**Table 5:** The concentration of elements in the leached solution before and after the adsorption process.

Element	Before adsorption (leached solution) (mg/L)	After adsorption (effluent) (mg/L)
Th	34.56	0.155
Ce	293.74	207.40
La	121.93	87.99
Nd	123.74	77.66
Pr	35.11	23.93
Sm	13.92	9.93
Eu	2.62	1.90
Gd	6.66	5.84
Tb	0.58	0.47
Dy	1.41	1.04

Adsorption conditions: 24 h contact time, room temperature, (2 ml) of the leach solution, pH (3) and (0.03 g) of PZ.

**Figure 18:**
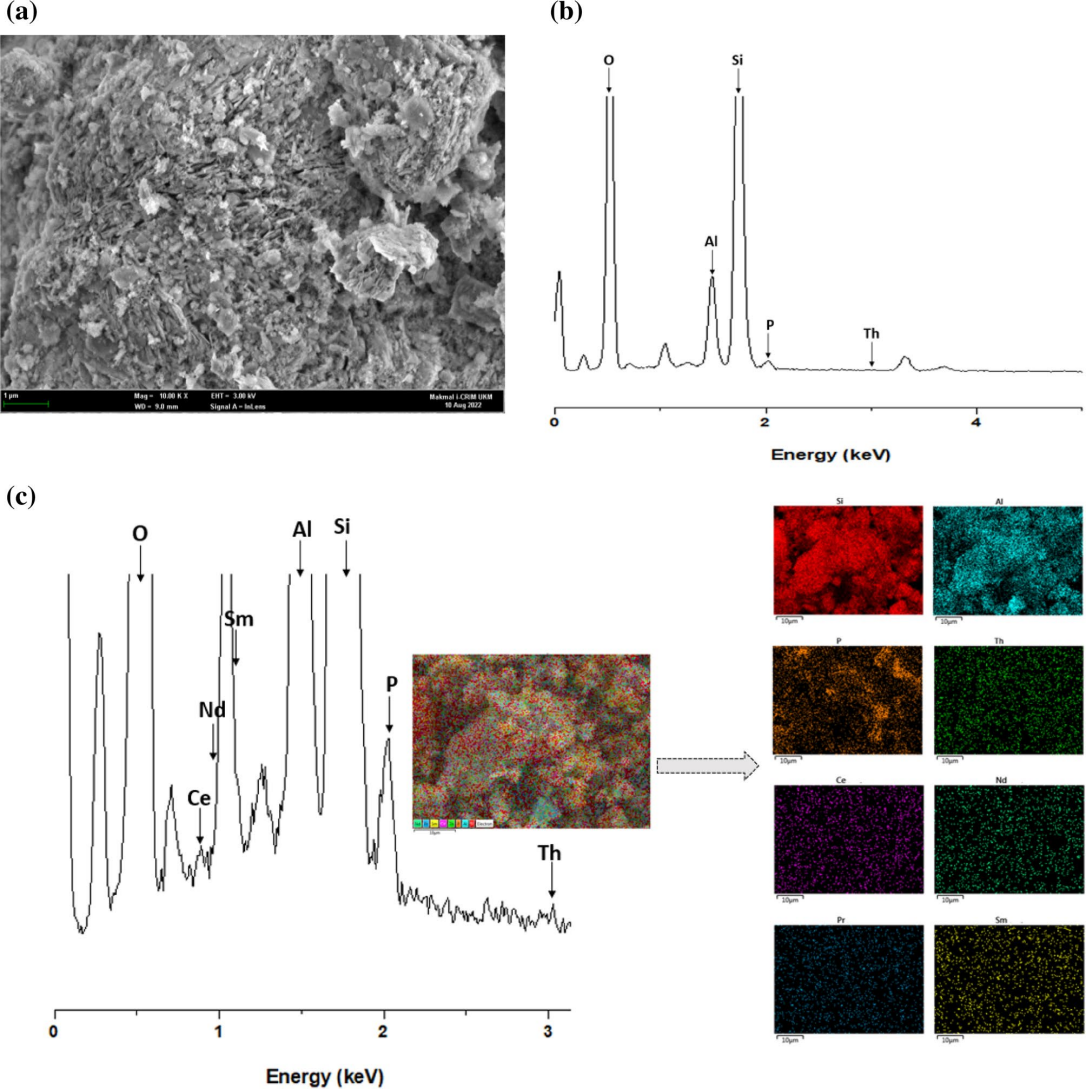
(**a**) The FESEM image of PZ after the adsorption of thorium from the leached solution of rare earth residue, at a magnification of 10000x; (**b**) the Full EDX spectrum of PZ after the adsorption process and (**c**) Close-up view of EDX spectrum and dot mapping showing the detection of thorium and some rare earths.

After the adsorption process of thorium ions from the leached solution of radioactive residue by PZ, the spent adsorbent was subjected to further FESEM–EDX analyses in order to verify the presence of thorium ions on the adsorbent material. The surface morphology of PZ adsorbent after the adsorption process is shown in Fig. 18a. In the FESEM image, the bright areas might refer to the accumulation of thorium and the rare earth elements on PZ surface. The EDX pattern of PZ adsorbent after the adsorption process is shown in Fig. 18b. The obtained results show that the EDX was capable of detecting thorium as well as the rare earth elements, but their concentrations are significantly low compared to oxygen, aluminum and silicon. Due to this fact, they cannot be seen clearly in the full pattern. However, in the close-up view as shown in Fig. 18c, thorium was observed at around 3 keV, and the rare earth elements were detected at the energy levels ranging from 0.8 to 1.1 keV. Additionally, the EDX elemental mapping results show that the thorium ions and rare earth elements are randomly adsorbed on the surface of the adsorbent material as demonstrated on Fig. 18c.

### The significant impact of thorium removal from rare earth industrial residue.

The reutilization of used adsorbents and the retrieval of metals are crucial considerations in adsorption separation processes. Adsorption is a well-established technology that minimizes the generation of secondary waste. However, the proper disposal of used adsorbents remains a significant challenge. Exploring the reuse and metal recovery from used adsorbents can provide a solution to this issue^88^. Although there have been several studies on recycling used adsorbents and recovering metals, limited research has focused on the disposal of used adsorbents. In our study, we observed that the phosphate-modified zeolite (PZ) adsorbent became saturated with thorium after the adsorption process. To comply with the guidelines of the International Atomic Energy Agency (IAEA), materials containing radionuclides must undergo a conditioning process prior to disposal. Typically, this process involves immobilizing the materials in containers, such as 200-L steel drums^89,90^. Therefore, the phosphate-modified zeolite (PZ) adsorbent will be promptly disposed of following the adsorption separation process, in accordance with the IAEA guidelines.

Malaysia’s rare earth (RE) industry has generated an average of 1.08 million tons of waste since its operation in 2015^91^. It requires 4 556,000 of 200 L disposal drums to store the end product waste in long-term disposal facilities. The estimated number of drums storing the RE industry’s radioactive residue were calculated using Eqs. (12) and (13) as follows^11^:

12 $$\mathrm{The }\,{V}_{R} \left(gal\right)={T}_{R} \left(tons\right) \times CF,$$

where ${V}_{R}$ represents volume of waste (in gallon) while ${T}_{R}$ and CF represents the mass of radioactive residue (in ton) and conversion factor tons of wet sludge to gallon respectively.

13 $${D}_{WT}=\frac{ {V}_{R} (gal)}{{V}_{SD} (gal)},$$

where ${D}_{WT}$ denotes of steel drum required to disposed waste without treatment while ${V}_{R}$ and ${V}_{SD}$ denotes volume of waste (in gallon) and volume of steel drum (in gallon) respectively.

As previously discussed, the modified clinoptilolite adsorbent in this study can remove 17.3 g of thorium for every 1 kg of adsorbent used, the highest capacity achieved in this study. In order to remove 1557 tons of thorium from 1.08 million tons of generated rare-earth residue, in relation to RE industry facilities, it requires approximately 90,559 tons of clinoptilolite adsorbent, which was calculated using Eq. (14)^11^:

14 $${m}_{ads}= \frac{{m}_{Th}}{{C}_{ads}},$$

where ${m}_{c}$ denotes mass of adsorbent required to remove thorium (in ton) while ${m}_{Th}$ and ${C}_{ads}$ represents the mass of thorium inside the radioactive waste (in kg) and specific adsorbent capacity (kg Th/tons adsorbent) respectively.

The total amount of disposal drum required in storing the modified clinoptilolite adsorbent post the adsorption process is approximately 229,000 drums which was calculated using Eqs. (15) and (16)^11^:

15 $${V}_{ads} \left(gal\right)={T}_{ads} \left(tons\right) \times CF,$$

where ${V}_{R}$ represents volume of adsorbent (in gallon) while ${T}_{R}$ and CF represents the mass of adsorbent (in ton) and conversion factor tons of adsorbent to gallon respectively.

16 $${D}_{AT}=\frac{ {V}_{ads} (gal)}{{V}_{SD} (gal)},$$

where ${D}_{AT}$ represents the number of steel drum required to disposed modified clinoptilolite adsorbent after adsorption treatment while ${V}_{ads}$ and ${V}_{SD}$ denotes volume of adsorbent (in gallon) and volume of steel drum (in gallon) respectively.

Reduction percentage of waste after adsorption treatment was calculated based on Eq. (17)^11^. It is clearly elucidated that the volume of waste would be reduced up to 95% yearly through the additional employment of adsorption treatment prior to storage. In this study, the phosphate-modified zeolite (PZ) adsorbent material will be disposed of in a steel drum for ultimate disposal. Therefore, further investigations into thorium recovery from PZ are not within the scope of this investigation. However, future research will explore the recovery of thorium and the potential reuse of PZ adsorbent for nuclear applications.

17 $$waste\,\, reduction (\%)= \frac{{D}_{WT}-{D}_{AT}}{{D}_{WT}} \times 100,$$

where *%* denotes reduce percentage volume steel drum being disposed while ${D}_{WT}$ *and* ${D}_{AT}$ represents the number of steel drum required to disposed waste without treatment and number of steel drum required to disposed adsorbent after adsorption treatment respectively.

## Conclusions

In this study, we aimed to enhance the removal of thorium using natural zeolite (clinoptilolite) modified with phosphate. The modified zeolite was thoroughly characterized using various techniques. To determine the optimal conditions for thorium adsorption, a series of experiments were conducted. The optimized conditions involved the use of 0.03 g of phosphate-modified zeolite (PZ) at a pH of 3, with shaking at 775 rpm for 24 h at room temperature (25 °C). Through the modification process using phosphate, the adsorption capacity of the natural zeolite was significantly improved, resulting in a maximum adsorption capacity of 17.3 mg/g for thorium.

The adsorption equilibrium was analyzed using the Langmuir, Freundlich, and Dubinin-Radushkevitch (D-R) models. Among these models, the Langmuir model provided the best fit for the experimental data within the studied concentration range at 25 °C, indicating its suitability for describing the adsorption process. Additionally, the kinetic studies revealed that the adsorption of thorium ions onto the phosphate-modified zeolite followed the pseudo-second-order model. The ultra-high efficiency of the PZ adsorbent in removing thorium makes it suitable for application in the removal of thorium from rare earth residue. Our results demonstrated that PZ exhibited the highest selectivity for thorium and achieved nearly complete removal of thorium ions. As a result, the use of PZ in thorium removal from rare earth residue is expected to reduce the waste volume for disposal by 95%. Overall, the successful removal of thorium and the potential reduction in rare-earth residue volume have positive implications for the rare earth sector in Malaysia. These findings are likely to foster favorable perceptions and attitudes towards the industry, ultimately contributing to its further development in the near future.

## Data Availability

The datasets generated and/or analysed during the current study are not publicly available due privacy or ethical restrictions but are available from the corresponding author on reasonable request.
